# A System for Discovering Novel Uricosurics Targeting Urate Transporter 1 Based on In Vitro and In Vivo Modeling

**DOI:** 10.3390/pharmaceutics16020172

**Published:** 2024-01-25

**Authors:** Xuechen Li, Chufan Qi, Mengjie Shao, Yajun Yang, Yuying Wang, Jiang Li, Zhiyan Xiao, Fei Ye

**Affiliations:** 1 Beijing Key Laboratory of New Drug Mechanisms and Pharmacological Evaluation Study, Institute of Materia Medica, Chinese Academy of Medical Science and Peking Union Medical College, Beijing 100050, China; 2 Diabetes Research Center, Chinese Academy of Medical Sciences and Peking Union Medical College, Beijing 100050, China; 3 Beijing Key Laboratory of Active Substance Discovery and Druggability Evaluation, Institute of Materia Medica, Chinese Academy of Medical Sciences and Peking Union Medical College, Beijing 100050, China

**Keywords:** hyperuricemia, urate transporter 1, uricosurics, uric acid, inhibitor

## Abstract

Hyperuricemia has become a global burden with the increasing prevalence and risk of associated metabolic disorders and cardiovascular diseases. Uricosurics act as a vital urate-lowering therapy by promoting uric acid excretion via the kidneys. However, potent and safe uricosurics are still in urgent demand for use in the clinic. In this study, we aimed to establish in vitro and in vivo models to aid the discovery of novel uricosurics, and to search for potent active compounds, especially targeting urate transporter 1 (URAT1), the major urate transporter in the kidney handling uric acid homeostasis. As a result, for preliminary screening, the in vitro URAT1 transport activity was assessed using a non-isotopic uric acid uptake assay in hURAT1-stably expressed HEK293 cells. The in vivo therapeutic effect was evaluated in a subacute hyperuricemic mouse model (sub-HUA) and further confirmed in a chronic hyperuricemic mouse model (Ch-HUA). By utilizing these models, compound CC18002 was obtained as a potent URAT1 inhibitor, with an IC_50_ value of 1.69 μM, and favorable uric acid-lowering effect in both sub-HUA and Ch-HUA mice, which was comparable to that of benzbromarone at the same dosage. Moreover, the activity of xanthine oxidoreductase, the key enzyme catalyzing uric acid synthesis, was not altered by CC18002 treatment. Taken together, we have developed a novel screening system, including a cell model targeting URAT1 and two kinds of mouse models, for the discovery of novel uricosurics. Utilizing this system, compound CC18002 was investigated as a candidate URAT1 inhibitor to treat hyperuricemia.

## 1. Introduction

Hyperuricemia is a chronic metabolic disease typically characterized by elevated concentrations of uric acid in the blood (420 µM in males and 360 µM in females). With the slightly acidic property (pKa1 = 5.75, pKa2 = 10.3) and weak solubility of uric acid, hyperuricemia is the leading risk factor for the development of gout, which is a form of osteoarthritis resulting from the abnormal deposition of excessive monosodium urate crystals in the osteoarticular and non-osteoarticular cavities, followed by the activation of acute aseptic inflammation and excruciating pain [1]. Moreover, emerging evidence has also demonstrated that hyperuricemia is closely associated with the progression of metabolic syndrome [2], cardiovascular diseases [3], chronic kidney disease (CKD) [4,5], etc. The report from the US National Health and Nutrition Examination Survey revealed that the prevalence of hyperuricemia has reached 19.1% from 1988 to 1994, 25.1% from 2007 to 2008, and 20% from 2015 to 2016 [6]. Notably, the prevalence of hyperuricemia has been rapidly increasing throughout the world, together with the growing burden of obesity and aging [7]. Therefore, early clinical interventions to prevent hyperuricemia are of great clinical importance.

Uric acid is the terminal product of purine metabolism in primates, such as humans and apes. Exogenous and endogenous purines are primarily synthesized into uric acid in the liver. In physiological conditions, the liver produces about 300–400 mg of uric acid per day. The uric acid is distributed throughout the bloodstream, and is excreted through the kidney and gut, in an amount of approximately 750 mg per day [8]. Both decreased excretion and overproduction of uric acid could disrupt uric acid homeostasis, and thus contribute to hyperuricemia. To modulate the synthesis of uric acid, xanthine oxidoreductase (XOR) in the liver is the key target that catalyzes the conversion from hypoxanthine to xanthine, and subsequently from xanthine to uric acid [9]. Several anti-hyperuricemic drugs acting as specific XOR inhibitors have been launched and are still utilized in clinical practice to treat hyperuricemia. However, side effects limited the use of several XOR inhibitors. The current first-line XOR inhibitor is allopurinol, which has been used clinically for over 50 years [10].

However, not all hyperuricemic patients are sensitive to XOR inhibitors. About two-thirds of uric acid is known to be excreted through the kidney, while the another one-third is excreted through the gut [11]. Notably, the kidney is the principal organ for the excretion of uric acid, and patients with hyperuricemia associated with chronic kidney disease showed significant deterioration in renal function, contributing to abnormal uric acid homeostasis [12]. Furthermore, impaired renal uric acid excretion contributes to clinically sustained hyperuricemia with an incidence of nearly 90% [13]. Prospective studies have shown that the efficacy of XOR inhibitors was remarkably limited, especially in patients with primary hyperuricemia not combined with CKD, in which uricosurics have better performance [13,14].

Uricosurics have been developed as anti-hyperuricemic drugs by improving uric acid excretion. In the kidney, the excretion of uric acid involves several renal physiological processes and sophisticated regulation of functional proteins in the luminal membrane. The uric acid handling mechanism is known as the four-component system [15]. Urate in the blood flows through the afferent glomerular arteriole and is completely filtered into the glomerulus. The filtered urate reaches the proximal tubule S1 and is subsequently reabsorbed by the epithelial cells. Continually, the reabsorbed urate is secreted by the proximal tubule S2, accounting for around 50% of the filtered load. Ultimately, approximately four-fifths of secreted urate is eventually reabsorbed at a post-secretory site in proximal tubule S3, while only about 10% of the total urate is eliminated from the body through urine. Notably, almost 90% of the uric acid filtered through the renal glomerulus is reabsorbed from the renal tubules into the bloodstream by several urate transporters, which are therefore strongly involved in the handling of blood uric acid levels [16].

Functional urate reabsorbing transporters in the kidney consist of the organic anion transporters family (OATs) and glucose transporter 9 (GLUT9). Urate transporter 1 (URAT1) is a member of OATs, with a characteristic structure consisting of 12 transmembrane domains. Located at chromosome 11q13.1, it has 555 amino acid residues and is encoded by the solute carrier family 22 member 12 (SLC22A12) [17]. With a restricted expression within the epithelial cells in the apical membrane of renal proximal tubules [18], URAT1 is an integral membrane protein mediating the exchange of extracellular urate with intracellular organic anions, such as lactate anions, and inorganic anions, such as chloride ion. Therefore, urate is reabsorbed from the lumen of the tubule into the epithelial cells. This process of reabsorption is not specific to a single substrate and displays affinity towards a broad range of organic anion substrates. Moreover, the motive force for URAT1-mediated absorption mainly depends on the ionic concentration gradient on both sides of the lumen, and the accumulation of extracellular organic anions, such as lactate, acetoacetate, hydroxybutyrate, and succinate, could disrupt the functional exchange of substrate and impede the urate reabsorption. URAT1 exhibits a profound capacity to maintain uric acid homeostasis in humans, mediating the majority of uric acid reabsorption in the kidney, and thus the dysfunction of URAT1 could lead to several uric acid-associated diseases. The rs475688 polymorphism of SLC22A12 is associated with the susceptibility to gout [19]. The loss-of-function mutation in the URAT1 gene, which could disrupt the function of URAT1 and hinder uric acid reabsorption, results in renal hypouricemia type 1, whose plasma uric acid level is generally below 60 μM (1 mg/dL) and uric acid excretion is close to 100%. Remarkably, mouse URAT1 displays 74% homology with human URAT1, and it has a relatively low affinity for urate (Km = 371 μM) compared to that of human URAT1 (Km = 1200 μM) [20]. Knockdown of the mouse SLC22A12 gene may lead to a mild rise in urinary uric acid excretion without a significant reduction in serum uric acid [21]. Different from URAT1, which mainly recognizes urate as the substrate, glucose transporter 9 (GLUT9) mediates the transport of urate as well as fructose [22]. GLUT9 is located in both the apical and basolateral membrane of proximal tubules, reabsorbing intraluminal urate into the tubular cells, and intracellular urate back to the interstitium, respectively. About 5% of the uric acid variance could be attributed to the GLUT9 mutation [23]. The expression and function of GLUT9 exhibit specificity across species. In mice, GLUT9 is expressed in both the distal and proximal tubules, which plays a multifaceted role in regulating uric acid homeostasis within the kidney.

Current clinically used uricosurics, including benzbromarone, probenecid, lesinurad, and dotinurad all have significant inhibitory effects on URAT1. The half maximal inhibitory concentration (IC_50_) value of benzbromarone against URAT1 is 0.3 μM [24]. Besides URAT1, benzbromarone and probenecid also down-regulated the expression of GLUT9. However, benzbromarone has been largely withdrawn from the European market, due to severe hepatotoxicity reports [25]. Moreover, the short half-life limited the use of probenecid [26]. Lesinurad, a selective URAT1 inhibitor with an IC_50_ value of 3.5 μM launched by AstraZeneca [27], was the initial FDA-approved antihyperuricemic drug targeting the URAT1. However, it has been withdrawn from the US market since 2019, shortly after being launched in 2015 [28]. Dotinurad was the latest URAT1 inhibitor, which was launched only in Japan in 2020. Although few studies regarding its clinical utility and safety have been reported [29], more information is still required. Among several selective URAT1 inhibitors under clinical trials, SHR4640, developed by Hengrui Pharma, is one of the fastest, with an IC_50_ value against hURAT1 of 33.7 nM [30]. Novel URAT1 inhibitors are still in urgent need of being successfully developed.

In this study, we aimed to establish in vitro and in vivo models, especially for screening potent uricosurics targeting URAT1 and obtaining active compounds. An experimental basis for the development of novel uricosurics has been provided.

## 2. Material and Methods

### 2.1. Chemicals and Reagents

To induce hyperuricemia, oteracil potassium (OP) (Meilunbio, Dalian, China), yeast extract (Oxoid, Hampshire, UK), and uric acid (Shanghai Yuanye Biotechnology, Shanghai, China) were purchased. Benzbromarone was from Excella GmbH (Feucht, Germany). Febuxostat was from TCI (Tokyo, Japan). SHR4640 and compound CC18002 were synthesized by Professor Zhiyan Xiao’s lab at the Institute of Materia Medica, CAMS & PUMC. For the measurement of in vivo experimental parameters, the kit for detecting blood uric acid was purchased from Biosino (Beijing, China), and the kit to determine plasma XOR activity was from Nanjing Jiancheng Bioengineering Institute (Nanjing, China). For the in vitro experiment, Krebs-Ringer buffer (Solarbio, Beijing, China) and uric acid kits (abcam, Cambridge, UK) were purchased. Milk-derived XOR, xanthine, and DMSO were purchased from Sigma-Aldrich (Darmstadt, Germany).

### 2.2. Animals

Male ICR mice weighing 20–24 g and male Kunming mice weighing 12–16 g were both purchased from Vital River Laboratory Animal Technology (Beijing, China) and housed in a constant environment with a temperature from 22 °C to 24 °C and humidity from 40% to 60%. The light/dark cycle was switched every 12 h. Free access to food and water was allowed at all times.

### 2.3. Cell Culture

Human embryonic kidney (HEK293) cells were cultured in the incubator at 37 °C with 5% CO_2_, supplemented with DMEM medium containing 10% fetal bovine serum, as well as 50 U/mL penicillin and streptomycin. URAT1 over-expressed HEK293 (URAT1-HEK293) cells were constructed as reported previously [31] and cultured in the same condition as the HEK293 cells.

### 2.4. Uric Acid Uptake Assay

HEK293 cells and URAT1-HEK293 cells were prepared in 24-well plates with around 2.5 × 10^5^ cells per well, and the uric acid uptake assay was applied when cells were around 80% confluent. URAT1-HEK293 cells were pretreated with compounds at various concentrations for 30 min. Both URAT1-HEK293 cells and HEK293 cells were then incubated with 750 μM uric acid buffer (dissolved in Krebs-Ringer buffer, pH 8.0) to allow uric acid uptake. After 30 min, cells were washed with cold PBS buffer and then collected to measure the intracellular uric acid level, following the manufacturer’s instructions. Briefly, 50 μL cell suspension and 50 μL reaction buffer were added to a black 96-well plate with a clear bottom and incubated away from light at 37 °C for 30 min. The intracellular uric acid level was measured using the fluorometric method. Protein concentration was then measured using BCA kits to normalize the fluorescence intensity. The amount of uric acid uptake by HEK293 cells was used as a blank value to exclude the intracellular uric acid uptake through the URAT1-unrelated method. IC_50_ value was eventually analyzed using the equation of log (inhibitor) vs. response-variable slope (four parameters) in Graphpad Prism 8.

### 2.5. Hyperuricemic Mice Models

To induce subacute hyperuricemia (sub-HUA), ICR mice were administrated with uric acid (200 mg/kg, i.p.) and OP (200 mg/kg, i.p.) at 0 h, followed by another OP administration (200 mg/kg, i.p.) at 2 h. Drugs were orally given at 1 day and 2 h prior to the first induction. Tail blood samples were collected at 0, 4, 6, 8, 10, 12, and 24 h to determine blood uric acid level. The uric acid–time curve was obtained, and the area under the curve (AUC_0–24h_) was subsequently calculated.

To induce chronic hyperuricemia (Ch-HUA), Kunming mice were fed with a special feeding chow containing yeast extract (20%) and OP (5%) and observed for 14 days. For pharmacological evaluation, drugs were orally administrated for 7 consecutive days from day 3. Negative control mice received equivalent vehicles. Blood uric acid level was monitored by collecting tail blood samples 6 h after drug administration. At day 10, mice were intraperitoneally injected with 1 mg/mL heparin and then euthanized. Whole blood samples were collected for further analysis.

### 2.6. Determination of Biochemical Parameters

To determine blood uric acid level, tail blood (20 μL) was collected and mixed with acetone solution (65 μL) (acetone/saline = 9:4), followed by centrifugation at 10,800 rpm for 7 min. The supernatant was then collected for uric acid measurement, according to the manufacturer’s instructions.

Plasma was collected after the whole blood was centrifuged at 3000 rpm for 4 min. Plasma XOR activity was measured using the commercial kits, under the manufacturers’ instructions.

### 2.7. Evaluation of XOR Activity In Vitro

XOR activity was assessed by measuring XOR-catalyzed uric acid. XOR (3 U/L) was co-incubated with 0.1% DMSO as a control group or different doses of compounds in PBS buffer (50 mM potassium phosphate, 0.1 mM EDTA, pH 7.8) in a 96-well plate. Xanthine (50 μM), the substrate, was added to a final volume of 250 μL to start the reaction at 37 °C for 20 min. The synthesized uric acid was determined by the absorbance read at 293 nm. Taking the uric acid production in the control group as 100%, the inhibition rates of the compounds were calculated.

### 2.8. Statistical Analysis

All data were presented as mean ± SD. All data conformed to the normal distribution assessed using the D’Agostino–Pearson test or Shapiro–Wilk test when small sample sizes were applied. One-way ANOVA was used for statistical analysis among multiple groups using the software Graphpad Prism 8. A *p* value of less than 0.05 was considered significant.

## 3. Results

Part I: In vitro and in vivo modeling for the discovery of URAT1 inhibitor

### 3.1. Establishment of an In Vitro Model for URAT1 Inhibitor Preliminary Screening

**Figure 1:**
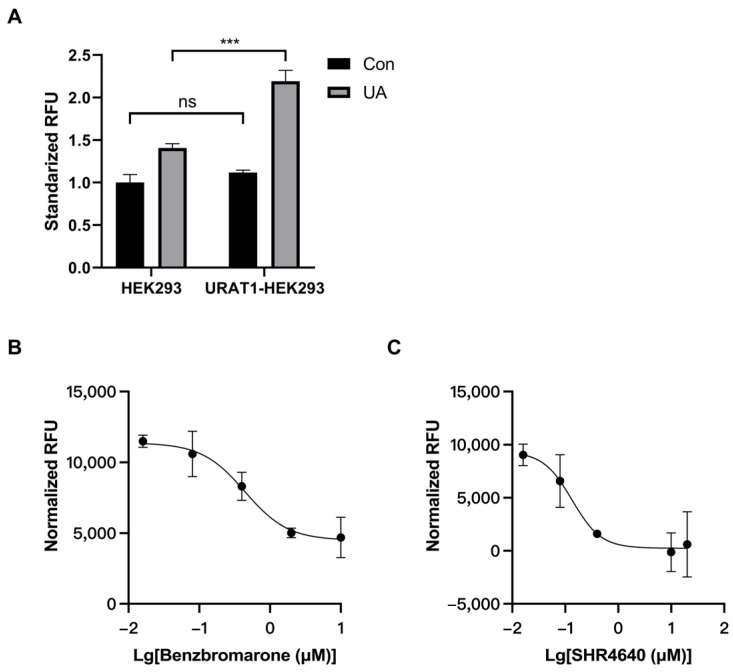
Assessing uric acid transport activity of URAT1 in cells. (**A**) The uric acid uptake was measured in both HEK293 cells (HEK293) and URAT1 over-expressed HEK293 cells (URAT1-HEK293). Cells were incubated in 750 μM uric acid buffer for 30 min, then collected for determination of intracellular uric acid level. Con, control group; UA, uric acid uptake group, n = 3. *** *p* < 0.001 between indicated groups; ns *p* > 0.05 between indicated groups. Different doses of (**B**) benzbromarone or (**C**) SHR4640 were pretreated in URAT1 over-expressed HEK293 cells for 30 min. The level of intracellular uric acid was determined and normalized by protein content. n = 3.

URAT1-HEK293 cells were applied to evaluate URAT1 activity, and HEK293 cells were used as the negative control. Intracellular non-isotopic uric acid uptake within 30 min in URAT1 over-expressed cells was remarkably higher than that in HEK293 cells (Figure 1A). Subtracting the uric acid uptake in HEK293 cells, the content of intracellular uric acid uptake in URAT1 over-expressed cells represented actual uric acid transport activity by URAT1. The IC_50_ value under multiple doses of drug treatment was fitted by Graphpad Prism 8. Using this method, the IC_50_ values against URAT1 activity of positive control drugs, benzbromarone, and SHR4640, were 0.44 μM and 0.13 μM, respectively (Figure 1B,C).

### 3.2. Establishing the Hyperuricemic Mouse Models for Pharmacological Evaluation

**Figure 2:**
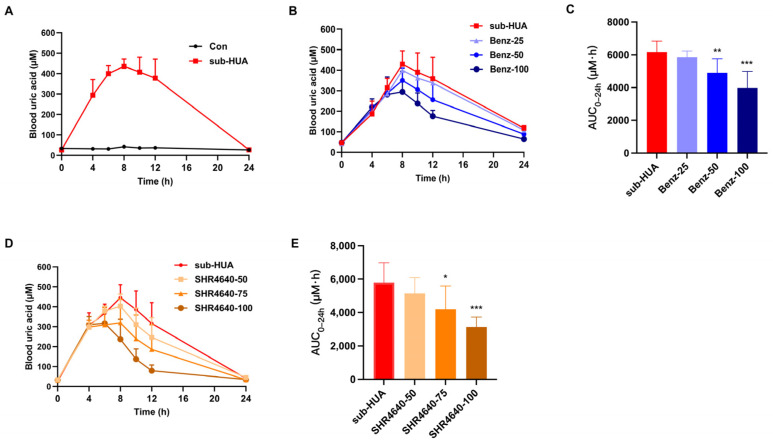
Characteristics of sub-HUA hyperuricemic mouse model. (**A**) After the mice received the first injection of uric acid and OP, blood uric acid level was measured at 0, 4, 6, 8, 10, 12, and 24 h, respectively. The levels of blood uric acid in the sub-HUA hyperuricemic mouse model (n = 9) are presented. In sub-HUA mice receiving 25, 50, and 100 mg/kg benzbromarone, respectively, (**B**) the blood uric acid–time curve and (**C**) the value of AUC_0–24h_ is presented (n = 10). In sub-HUA mice administrated with 50, 75, and 100 mg/kg SHR4640, respectively, (**D**) the blood uric acid–time curve and (**E**) AUC_0–24h_ was obtained (n = 8). * *p* < 0.05, ** *p* < 0.01, *** *p* < 0.001 vs. sub-HUA.

Hyperuricemic mouse models were established to evaluate the therapeutic effect of uricosurics targeting URAT1. The sub-acute hyperuricemic (sub-HUA) mice were induced by uric acid and OP, and maintained high blood uric acid level for about 24 h. After being induced by uric acid and OP for 4 h, the level of blood uric acid in sub-HUA mice was significantly elevated, peaked at about 8 h, and finally dropped to baseline at about 24 h (Figure 2A). By orally administrating benzbromarone at the dose of 25, 50, and 100 mg/kg, the peak uric acid levels at 8 h were decreased by 7.7%, 20.5%, and 35.4% (Figure 2B), and the AUC_0–24h_ was reduced by 7.7%, 20.5% and 35.4% (Figure 2C), respectively, compared to the model control sub-HUA group. Similarly, SHR4640 administration reduced the peak uric acid level by 9.5%, 28.2%, and 46.7% (Figure 2D), and decreased the AUC_0–24h_ value by 11.2%, 27.6%, and 45.9% (Figure 2E) at the dose of 50, 75 and 100 mg/kg, respectively.

**Figure 3:**
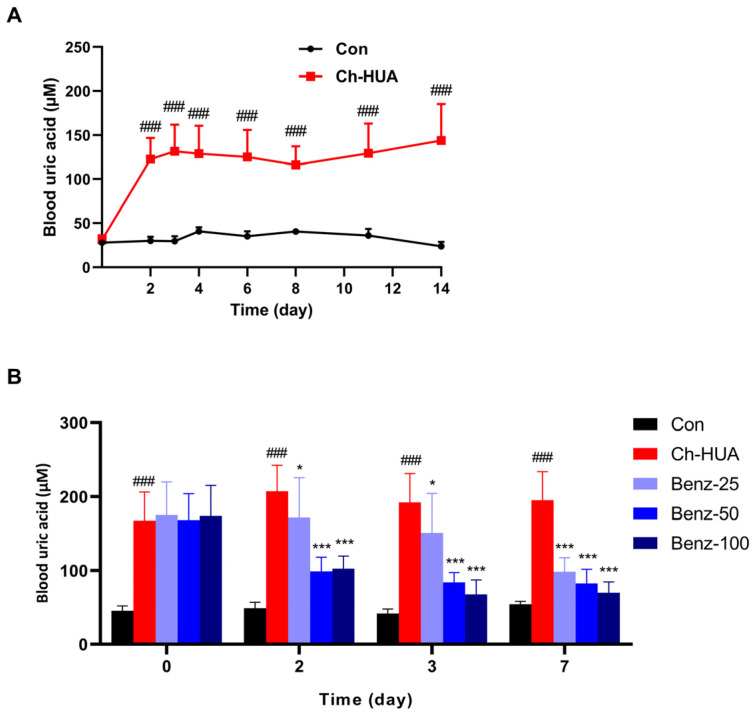
Characteristics of Ch-HUA hyperuricemic mouse model. (**A**) Uric acid–time curve in Ch-HUA model mice. n = 9. ### *p* < 0.001 vs. Con. (**B**) The uric acid level was measured before and after treatment for 2, 3, and 7 days by consecutive administration of 25, 50, or 100 mg/kg benzbromarone, respectively, by gavage, in Ch-HUA model mice. Con, normal control group; Ch-HUA, model control group. n = 10. ### *p* < 0.001 vs. Con; * *p* < 0.05, *** *p* < 0.001 vs. Ch-HUA.

To induce the chronic hyperuricemia (Ch-HUA) model, mice were constantly fed with a special purine-rich diet containing yeast extract and OP. Within 14 days of observation, the Ch-HUA mice exhibited steady hyperuricemia from day 3 (Figure 3A). In Ch-HUA model mice, benzbromarone daily administrated daily at a dose of 25, 50, or 100 mg/kg, respectively, for 7 consecutive days. The blood uric acid level was dose-dependently and time-dependently decreased (Figure 3B). During the treatment, the blood uric acid level in benzbromarone-treated Ch-HUA mice remained no lower than that in control mice (Con) for 7 days, which did not drop to an abnormal lower range. However, the inhibition of uric acid degradation by OP administration in benzbromarone-treated Ch-HUA mice should also be considered.

Part II: Discovery of novel uricosurics targeting URAT1

### 3.3. Discovery of URAT1 Inhibitory Compound In Vitro

**Figure 4:**
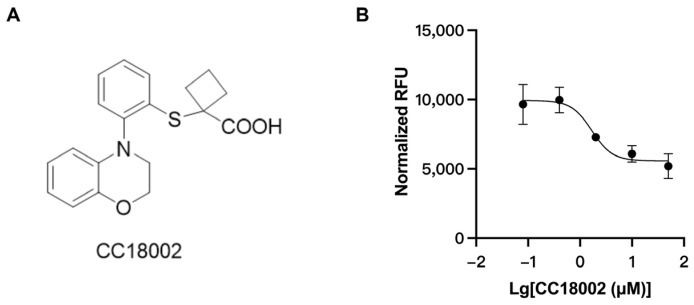
URAT1 inhibition by CC18002. (**A**) The chemical structure of compound CC18002. (**B**) The uric acid uptake normalized by protein content with co-incubation of compound CC18002 at multiple doses in URAT1 over-expressed cells is presented.

The in vitro uric acid uptake assay was applied for preliminary screening of a compound set, and CC18002 (Figure 4A) was obtained as an active compound. CC18002 exhibited potent URAT1 inhibitory activity, with an IC_50_ value of 1.69 μM (Figure 4B). The IC_50_ value provided by our uric acid uptake assay was a good match with the result from the transient expression system with URAT1-HEK293 cells and ^14^C labeled uric acid substrate.

### 3.4. The Uric Acid-Lowering Effect of CC18002 In Vivo

**Figure 5:**
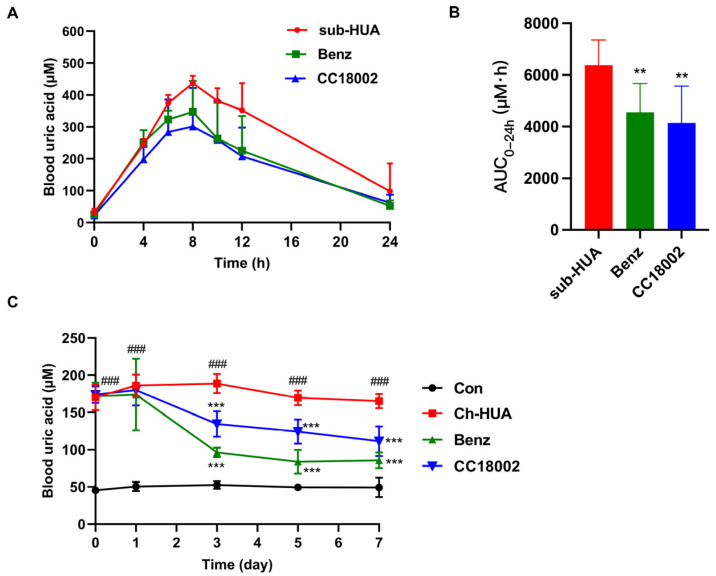
Therapeutic evaluation of the active compounds in both sub-HUA and Ch-HUA mouse models. (**A**) The uric acid–time curve and (**B**) AUC_0–24h_ were obtained in sub-HUA mice treated with benzbromarone or CC18002 at the same dose of 100 mg/kg, respectively. n = 9. ** *p* < 0.01 vs. sub-HUA. (**C**) Blood uric acid level was monitored in Ch-HUA model mice, which were orally administered with benzbromarone or CC18002 at the same dose of 50 mg/kg for 7 days. n = 10. ### *p* < 0.001 vs. Con; *** *p* < 0.001 vs. Ch-HUA.

To evaluate the uric acid-lowering effect of CC18002 in vivo, 100 mg/kg CC18002 and the positive control drug, benzbromarone, was orally administrated in sub-HUA mice. Compound CC18002 administration significantly decreased the peak level of blood uric acid by 30.6% at 8 h, which was greater than that of benzbromarone at the same dosage, by 20.1% (Figure 5A). CC18002, as well as benzbromarone treatment, both remarkably decreased the AUC_0–24h_ value (Figure 5B).

The effect of CC18002 on hyperuricemia was further confirmed in chronic Ch-HUA mice. Ch-HUA model mice were orally administered with benzbromarone or CC18002 at the same dose of 50 mg/kg. CC18002 treatment significantly ameliorated hyperuricemia from day 3 and gradually lowered the blood uric acid level until day 7, which was comparable to benzbromarone at the same dosage (Figure 5C).

### 3.5. CC18002 Had No Effect on XOR

**Figure 6:**
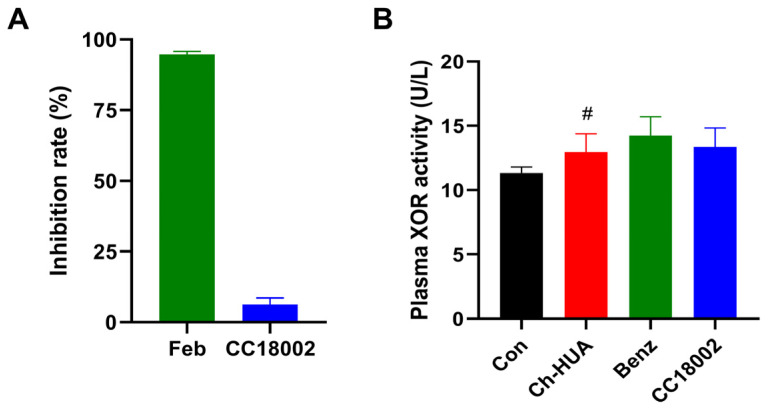
Effect of CC18002 on XOR activity. (**A**) Febuxostat (0.1 μM) or CC18002 (10 μM) was co-incubated with XOR (3 U/L) and xanthine (50 μM) at 37 °C for 20 min, and 0.1% DMSO, the solvent of febuxostat and CC18002, was co-incubated as control. The level of XOR-catalyzed uric acid was determined by the absorbance at 293 nm. The inhibition rate was then calculated. (**B**) Plasma XOR activity was measured in Ch-HUA mice treated with 50 mg/kg benzbromarone or CC18002. n = 10. # *p* < 0.05 vs. Con.

Finally, we examined the effect of CC18002, the active compound targeting URAT1, on XOR which is the key enzyme in uric acid synthesis. Firstly, we examined its inhibitory effect on XOR in vitro. Co-incubated with 0.1 μM febuxostat, the clinical XOR inhibitor, the XOR activity was almost completely inhibited, by 94.8% ± 1.1%. However, the XOR activity was barely inhibited, by 6.3% ± 2.2%, with co-incubation of 10 μM CC18002 (Figure 6A). Next, we evaluated the effect of CC18002 on XOR in vivo, where Ch-HUA model mice were used. As shown in Figure 6B, the plasma XOR activity was elevated in Ch-HUA model mice. Benzbromarone and CC18002 treatment for 7 days did not significantly alter plasma XOR activity in Ch-HUA mice. Our result indicated that CC18002 had no effect on XOR activity either in vivo or in vitro.

## 4. Discussion

In this study, we established an in vitro uric acid uptake model for the preliminary screening of novel URAT1 inhibitors, and two hyperuricemic mouse models with different pathophysiological characteristics to validate the therapeutic effect on hyperuricemia. By applying these models, we discovered compound CC18002 as a potent URAT1 inhibitor exhibiting strong URAT1 inhibitory activity in vitro and a favorable uric acid-lowering effect in both sub-HUA and Ch-HUA hyperuricemic mouse models, respectively.

The key technique in measuring URAT1 activity in vitro was to accurately detect the small amount of intracellular uric acid. The conventional uric acid uptake assay applied ^14^C-labelled uric acid and measured the amount of intracellular radiation by liquid scintillation counter to calculate URAT1 transport activity [32,33]. In this study, we applied a safer non-isotopic method by precisely measuring the intracellular uric acid at a relatively low level. The calculated IC_50_ value of the positive control drugs, benzbromarone, and SHR4640, was 0.44 μM and 0.134 μM, which was 1.5 fold and 3.6 fold of the reported value, respectively. These results indicated that the accuracy of this new non-isotopic cell model evaluating URAT1 activity in this study was comparable to the isotopic model reported previously [24]. Moreover, this non-isotopic model was more environmentally friendly and therefore advantageous for the applications in the future. Notably, the discovery of a selective URAT1 inhibitor still required the evaluation of its activity on other urate transporters, including GLUT9, ABCG2, OAT1, OAT3, etc. The combination of the effects on these urate transporters contributed to the final pharmacological effect of the compound. In this study, we did not exclude the effect of tested compounds on other urate transporters. Further studies will be required to confirm these effects.

The uric acid homeostasis was maintained by the proper synthesis and excretion of uric acid. Most hyperuricemic mouse models were induced by the substrate of XOR, which increased the production of uric acid and therefore elevated the blood uric acid level. Hypoxanthine [34,35,36,37,38], adenine [39,40], and inosine [41] were commonly used as the substrate to induce hyperuricemic mouse or rat models. Given that OP acts as a uricase inhibitor, preventing the breakdown of uric acid into allantoin and helping to maintain the high level of uric acid, it was usually administered together with the major inducer. However, these models were more suitable for the pharmacological evaluation of uric acid-synthesis targeted drugs.

To evaluate the therapeutic effect of uricosurics, hyperuricemic models directly induced by uric acid, the end product of purine metabolism, were designed. Previous work in our lab showed a hyperuricemic mouse model (UA-HUA) induced by 150 mg/kg uric acid (i.p., 0.1 mL/10 g body weight) and 150 mg/kg OP (i.p., 0.1 mL/10 g body weight). The blood uric acid level of these UA-HUA mice reached a peak level of over 200 μM at about 1 h, and then dropped back to baseline at about 4 h. Benzbromarone (100 mg/kg) and SHR4640 (100 mg/kg) were used as positive control and orally administrated 1 day and 2 h prior to the induction. However, although benzbromarone treatment significantly lowered the blood uric acid level and the AUC_0–4h_ value, SHR4640 treatment did not exert a uric acid-lowering effect during the 4 h observation. It was speculated that the hyperuricemic state should last longer and the peak blood uric acid level be higher, to allow enough time and space for URAT1 inhibitors to take effect. Therefore, in this study, the sub-HUA mouse model was designed. A higher dose of uric acid (200 mg/kg, i.p., 0.1 mL/10 g body weight) and OP (200 mg/kg, i.p., 0.1 mL/10 g body weight) was firstly injected at 0 h, and another 200 mg/kg OP (i.p., 0.2 mL/10 g body weight) was injected at 2 h to retard the degradation of uric acid, therefore maintaining the high level of uric acid for a longer time. As our results showed, the high level of blood uric acid, over 400 μM at peak level, lasted for almost 24 h in sub-HUA mice, allowing sufficient time for active compounds to lower blood uric acid level by promoting uric acid excretion. Effectively, the uric acid-lowering effect of benzbromarone and SHR4640 was observed from 6 h in sub-HUA mice. Therefore, it was speculated that the false-negative rate of discovering active uricosurics should be lower in the sub-HUA mouse model than in other hyperuricemic models that were maintained for a shorter time. The sub-HUA mouse model was characterized as uric acid-induced severe hyperuricemia lasting for almost 24 h and easily operated, which was suitable for pharmacological evaluation of uric acid-excretion targeted drugs.

Although patients had a transient high level of blood uric acid occasionally, chronic hyperuricemia more commonly occurred in the clinic. Ch-HUA mice were induced by a freely accessed diet containing purine-rich yeast extract, therefore exerting a steady high level of blood uric acid for at least 14 days. The Ch-HUA mouse model was characterized as long-lasting and steady hyperuricemia, which was closer to clinical hyperuricemia. In this study, this model was used to confirm the therapeutic effect of active compounds and further evaluate its sustainable effect by multiple dosing. Apart from being utilized to evaluate the therapeutic effect of anti-hyperuricemic drugs, it might be further used to induce hyperuricemia-related metabolic disorders and be of more utilization in the mechanism studies.

In this study, we used an in vitro uric acid uptake assay for preliminary screening and obtained compound CC18002, exerting potent URAT1 inhibitory activity, with an IC_50_ value of 1.69 μM, which was comparable to that of benzbromarone. The uric acid-lowering activity of CC18002 in vivo was then evaluated in sub-HUA mice. CC18002 treatment markedly decreased blood uric acid level and the AUC_0–24h_ value, which was non-inferior to benzbromarone at the same dosage of 100 mg/kg. Furthermore, the therapeutic effect of CC18002 in Ch-HUA mice was gradually enhanced during the administration period, similar to that of benzbromarone at the same dosage of 50 mg/kg. Finally, we excluded the possible effect of CC18002 on XOR, the key enzyme mediating uric acid synthesis. Our result indicated compound CC18002 to be a potent URAT1 inhibitor and an excellent candidate for hyperuricemia treatment.

## 5. Conclusions

Targeting URAT1, a system for the discovery of novel uricosurics was established, including the cell model based on the non-isotopic microscale uric acid detection technique in URAT1-overexpressed HEK293 cells, the sub-HUA mouse model characterized as transient severe hyperuricemia, and the Ch-HUA mouse model characterized as chronic steady hyperuricemia. By applying these in vitro and in vivo models, we found compound CC18002 to be a potent URAT1 inhibitor with favorable URAT1 inhibitory activity and uric acid-lowering effects. Our result supported CC18002 being further developed to treat hyperuricemia.

## Data Availability

Data contained within the article.
